# Uncovering Molecular Mechanisms of Feed Efficiency in Pigs Through Multi-Omics Analysis of the Jejunum

**DOI:** 10.3390/ani15020137

**Published:** 2025-01-08

**Authors:** Saixian Zhang, Yue Xiang, Yaobang Jian, Qiulin Zhao, Jiahui Sun, Yi Huang, Jing Xu, Xiaolong Qi, Jingjin Li, Zhuqing Zheng, Liangliang Fu, Yuwen Liu, Xinyun Li

**Affiliations:** 1 Shenzhen Branch, Guangdong Laboratory for Lingnan Modern Agriculture, Key Laboratory of Livestock and Poultry Multi-Omics of MARA, Agricultural Genomics Institute at Shenzhen, Chinese Academy of Agricultural Sciences, Shenzhen 518124, China; zhangsaixian@caas.cn (S.Z.); qixiaolong@caas.cn (X.Q.); lijingjin@caas.cn (J.L.); 2 Key Laboratory of Agricultural Animal Genetics, Breeding and Reproduction of Ministry of Education and Key Laboratory of Swine Genetics and Breeding of Ministry of Agriculture, College of Animal Science and Technology, Huazhong Agricultural University, Wuhan 430070, China; yxiang@webmail.hzau.edu.cn (Y.X.); yaobangjian@webmail.hzau.edu.cn (Y.J.); qiulinzhao@webmail.hzau.edu.cn (Q.Z.); sun_jiahui314@webmail.hzau.edu.cn (J.S.); huang_yi@webmail.hzau.edu.cn (Y.H.); jingxu1874@webmail.hzau.edu.cn (J.X.); zzq_1207@mail.hzau.edu.cn (Z.Z.); fuliangliang2017@mail.hzau.edu.cn (L.F.)

**Keywords:** feed efficiency, pigs, jejunum, RNA-seq, ATAC-seq, lipid metabolism, immune response

## Abstract

Feed efficiency (FE) is a crucial trait in pig production that influences both economic viability and environmental sustainability. The jejunum, an essential organ for nutrient absorption, plays a significant role in determining FE by affecting how pigs process and utilize feed. To explore the genetic and regulatory mechanisms behind FE, we conducted an integrative multi-omics study using RNA sequencing (RNA-seq) and ATAC sequencing (ATAC-seq) on pigs with high and low FE. By comparing gene expression and chromatin accessibility profiles in the jejunum, we identified key differentially expressed genes (DEGs) and differentially accessible regions (DARs) associated with lipid metabolism and immune function, both of which are critical pathways for efficient growth. Notably, we identified transcription factors such as GATA4 and EHF and genes like *SCARB1* and *GRXCR1* that may play regulatory roles in FE. Our findings provide novel insights into the molecular mechanisms governing FE in pigs, offering potential targets for genetic selection and nutritional interventions to enhance feed efficiency and sustainability in pig production.

## Simple Summary

Feeding pigs efficiently is crucial for both farmers and the environment. The small intestine, particularly the jejunum, plays a key role in absorbing nutrients from food. Our study aimed to understand the genes and cellular processes involved in efficient feed use in pigs. We compared the activity of genes and the accessibility of their DNA in the jejunum of pigs with high and low feed efficiency. This revealed genes and chromatin regions potentially linked to efficient feed use. Additionally, we identified important molecules that might control these genes. These findings offer new insights for enhancing pig farming practices, making them more cost-effective and environmentally friendly.

## 1. Introduction

Feed efficiency (FE) is a critical indicator in modern pig farming, reflecting the growth rate and feed utilization of pigs [1]. Improving FE not only significantly reduces production costs but also mitigates environmental pollution and greenhouse gas emissions, making it a key strategy for achieving sustainable agriculture [1,2]. As the global livestock industry faces challenges such as limited resources, environmental pressures, and shifting market demands, optimizing FE has become increasingly important [3]. Consequently, a deeper understanding of FE and its improvement is crucial for boosting the economic benefits of farming, promoting green agriculture, and ensuring food security.

Among the many factors influencing FE in pigs, the jejunum plays a pivotal role [4]. As a central component of the pig’s digestive system, the jejunum is primarily responsible for the absorption of vital nutrients such as proteins, fats, and carbohydrates [5,6,7]. Recent studies have highlighted that the digestive and absorptive functions of the jejunum are closely linked to feed conversion efficiency, directly impacting growth rate and feed utilization [8,9,10]. By investigating the genetic and molecular mechanisms underlying jejunal function, researchers can identify potential targets for improving FE through selective breeding and dietary interventions.

To gain deeper insights into the genetic basis of FE, we conducted an integrative multi-omics analysis, combining RNA sequencing (RNA-seq) and ATAC sequencing (ATAC-seq), on pigs with high and low FE. By comparing gene expression and chromatin accessibility profiles in the jejunum, we aimed to identify differentially expressed genes (DEGs) and differentially accessible regions (DARs) associated with FE. The integration of these datasets allowed us to construct a gene regulatory network, revealing key transcription factors and signaling pathways that may regulate FE in pigs. This study provides a comprehensive understanding of the molecular mechanisms underlying FE in pigs, offering valuable insights for developing strategies to improve FE and sustainability in pig production.

## 2. Materials and Methods

### 2.1. Collection of Jejunal Samples

All pigs were sourced from a commercial farm (Wuhan COFCO Meat Products Co., Wuhan, China) and maintained under identical feeding and housing conditions. To identify pigs with high and low FCR values, we selected two individuals with low FCR values (2.11 and 2.21, respectively) and two individuals with high FCR values (2.88 and 3.42, respectively) from a population of 484 pigs, which had a mean FCR value of 2.37 (Supplementary Table S1) [11]. At 180 days of age, the pigs were euthanized following an overnight fast in accordance with the approved animal care protocol (HZAUSW-2023-0023). Euthanasia was performed using a two-step procedure: initial sedation with an intramuscular injection of azaperone (4 mg/kg) and atropine (0.04 mg/kg), followed by anesthesia with an intramuscular injection of xylazine (4 mg/kg) and Zoletil (2 mg/kg). Jejunal samples were collected and immediately stored at −80 °C for subsequent molecular analyses. All animal procedures were approved by the Ethics Committee of Huazhong Agricultural University.

### 2.2. Generation of RNA-Seq Libraries

Total RNA was extracted from jejunal tissues using TRIzol reagent. RNA integrity was assessed by measuring the RNA Integrity Number (RIN), which was required to be more than 7. Subsequently, mRNA was purified using the VAHTS mRNA Capture Beads (Vazyme N401 kit, Vazyme, Nanjing, China). A strand-specific RNA-seq library was constructed using the Vazyme NR605 kit, which involved the following steps: first-strand cDNA synthesis, second-strand cDNA synthesis, adapter ligation, PCR enrichment, and purification using magnetic beads. The constructed strand-specific sequencing library was then sequenced on an Illumina NovaSeq 6000 platform (San Diego, CA, USA).

### 2.3. Analysis of RNA-Seq Data

TrimGalore (v0.6.6) (https://github.com/FelixKrueger/TrimGalore (accessed on 14 July 2024)) was first used to remove adapters and quality control with default parameters. The cleaned reads were then aligned to the pig susScr11 reference genome using STAR (2.5.1b) [12]. The genomic annotation file (GTF 101 version) from the Ensembl database was used to retrieve the genomic information, and promoter regions in this study were defined as the regions from 2500 bp upstream to 1000 bp downstream of the TSS. Gene expression levels were quantified using RSEM (1.2.31) to calculate TPM values [13]. Genes with at least 3 mapped reads in two samples were retained for further analysis. Differential expression analysis was performed using the DEseq2 package [14]. Genes with an absolute log2 fold change ≥ 0.58 and a *p*-value < 0.05 were considered differentially expressed. Volcano plots were generated using ggplot2 to visualize the differential gene expression between the high and low FE groups.

### 2.4. Generation of ATAC-Seq Libraries

Jejunal tissue samples were ground into fine powder in liquid nitrogen. Approximately 0.02 g of tissue was used for ATAC-seq library preparation. The tissue was suspended in 1 mL of pre-cooled DPBS and mixed by pipetting. Then, 1 mL of lysis buffer (50 mM HEPES, pH 7.5, 140 mM NaCl, 1 mM EDTA, pH 8.0, 10% glycerol, 0.5% NP-40, 0.25% Triton X-100) was added and mixed by pipetting. The mixture was incubated on ice for 10 min with gentle inversion. After centrifugation at 1000× *g* for 5 min at 4 °C, the supernatant was discarded. The cell pellet was resuspended in 1 mL of DPBS and filtered through a 70 μm cell strainer into a new tube. Trypan blue was added at a ratio of 1:4 to the cell suspension and mixed. The cells were counted using an inverted microscope. A total of 50,000 cells were collected and centrifuged at 500× *g* for 10 min at 4 °C, the supernatant was removed, and the cell pellet was resuspended in 50 μL of transposition mixture (25 μL 2 × TD buffer, 16.5 μL PBS, 0.5 μL 1% Digitonin, 0.5 μL 10% Tween-20, 5 μL H_2_O, 2.5 μL Tn5 enzyme). The mixture was gently mixed and incubated at 37 °C, 1000 rpm for 1 h in a thermomixer. After incubation, DNA was purified using the DNA Clean & Concentrator-5 Kit (TD413-50) from Zymo Research (Irvine, USA). For library amplification, the following reaction was performed: 10 μL 5 × TAB, 1 μL TAE, 2.5 μL each of Index 1 and Index 2, 14 μL nuclease-free ddH_2_O, and 20 μL DNA fragments. The PCR conditions were as follows: 72 °C for 3 min, 98 °C for 30 s, followed by 9 cycles of 98 °C for 10 s, 60 °C for 30 s, and 72 °C for 30 s, with a final extension at 72 °C for 5 min. The amplified DNA fragments were purified using KAPA Pure Beads (Roche, Basel, Switzerland) to obtain fragments of 150–800 bp for sequencing. Sequencing was then performed on an Illumina NovaSeq 6000 platform (San Diego, CA, USA).

### 2.5. Analysis of ATAC-Seq Data

Adapter sequences were trimmed from the raw ATAC-seq reads using TrimGalore (v0.6.6). The trimmed reads were then aligned to the pig susScr11 reference genome using Bowtie2 (v2.3.4.3) [15]. SAMtools (v1.9) and Picard (v2.20.7-0) (https://github.com/broadinstitute/picard (accessed on 14 July 2024)) were used to filter the BAM files, removing reads with low mapping quality (MAPQ < 30), unmapped reads, singletons, secondary alignments, and PCR duplicates [16]. Subsequently, BEDTools (v2.29.0) was used to remove reads mapped to the mitochondrial genome and convert the filtered BAM file into a BEDPE format, which was then extracted as tagAlign [17]. MACS2 (v2.2.4) was used to call peaks (−*p* 0.01 --nomodel --shift 75 --extsize 150 -B --SPMR --keep-dup all --call-summits), generating narrowPeak files [18]. NSC and RSC were calculated based on the ENCODE ATAC-seq analysis pipeline, and quality control was performed according to ENCODE standards. DEseq2 was used to identify differential peaks between groups based on the following criteria: absolute log2 fold change ≥ 0.58 and *p* < 0.05. For motif enrichment analysis, a genome index was built using the configureHomer.pl tool from HOMER [19]. The findPeaks command was used to find peaks in the ATAC-seq data, and the findMotifsGenome.pl command was used to identify enriched DNA sequence motifs within the peaks, revealing transcription factor binding sites.

### 2.6. Functional Annotation of Genes and Peaks

For the functional analysis of genes regulated by differential peaks and DEGs in the transcriptome, we utilized Ensembl Biomart to convert pig gene names to their human orthologs [20]. The resulting human gene IDs were then subjected to Gene Ontology (GO) enrichment analysis using Metascape to identify significantly enriched terms [21]. The results of GO enrichment analyses returned by Metascape include both *p*-values and enrichment scores. The enrichment score indicates the ratio of enrichment of genes in the target GO term within our gene list compared to the overall background gene set.

### 2.7. Construction of Gene Regulatory Network

To construct the gene regulatory network involved in FE, we first scanned DARs for motifs using FIMO from the MEME suite with reference motifs from the JASPAR2020 database [22,23]. Subsequently, the nearest gene approach was then applied to determine the genes regulated by these DARs. To refine our analysis, we filtered the transcription factors and their target genes based on three criteria: transcription factors must be DEGs, must have been previously linked to FE, and their target genes must also be DEGs. A gene regulatory network was then constructed using the filtered transcription factors and their target genes.

### 2.8. Validation of Transcription Factors and Genes in Gene Regulatory Network

The frozen jejunum tissue is ground to a powder in liquid nitrogen. Total RNA from the tissue powder was extracted using FastPure Complex Tissue/Cell Total RNA Isolation Kit (Vazyme, Nanjing, China) and reversely transcribed using the HiScript IV RT SuperMix for qPCR (+gDNA wipe) (Vazyme). Quantitative PCR was undertaken using the Taq Pro Universal SYBR qPCR Master Mix (Vazyme). Relative gene expression was normalized to glyceraldehyde-3-phosphate dehydrogenase (*GAPDH*) mRNA level with the 2^−∆∆Ct^ method. Statistical significance was determined by comparing the relative gene expression between high and low groups using the Wilcoxon rank-sum test. The primers for *IFIT2* and *GATA4* were as follows: *IFIT2* (forward: GCACAGCAATCATGAGTGAGAC, reverse: GGCCTGTATGTTGCACATCG) and *GATA4* (forward: GCAATGCGGAAAGAGGGGAT, reverse: GCACTAACTGAGAAGGTCTGGG).

## 3. Results

### 3.1. Identification of DEGs in the Jejunum Between Pigs with High and Low FE

**Figure 1:**
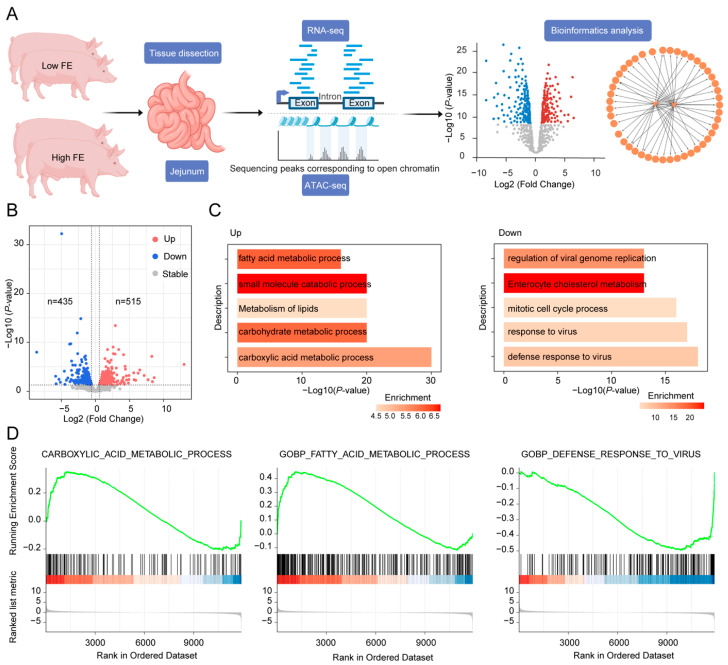
Differential expression analysis between high and low FE pigs. (**A**), Schematic representation of the sample dissection, experimental and bioinformatics analysis workflow used in this study. (**B**), Volcano plot showing differentially expressed genes (DEGs) between high and low FE pigs. “Up” and “Down” denote upregulated and downregulated genes, respectively, in high FE pigs compared to low FE pigs. The horizontal dashed line represents the threshold of raw *p*-value < 0.05, and two vertical dashed lines indicate the thresholds for upregulated (right, log2 fold change ≥ 0.58) and downregulated (left, log2 fold change ≤ −0.58) genes. (**C**), Functional annotation of upregulated and downregulated DEGs. (**D**), Gene Set Enrichment Analysis (GSEA) plots presenting three representative significantly enriched functional terms. The genes are ordered by fold change values.

To explore the gene expression differences in the jejunum between pigs with high and low FE, we collected jejunal tissue from four Large White pigs, two with high FE and two with low FE, and performed RNA sequencing (Figure 1A). After quality control, we obtained a total of 52.26 GB of high-quality data, with an average mapping rate of 85.34% to the reference genome (Supplementary Table S2). To assess the reproducibility of the data, we calculated the Spearman correlation coefficients between each pair of samples. As shown in Supplementary Figure S1A, the expression patterns across all samples were highly correlated (0.99~1.00), with an average correlation coefficient of approximately 0.99. Furthermore, the correlations within the high FE group and the low FE group were both significant (*p* < 2.2 × 10^−16^), with correlation coefficients of 0.98 (Supplementary Figure S1B).

We then conducted differential expression analysis between the high and low FE groups. In total, we identified 950 DEGs, of which 435 were downregulated, and 515 were upregulated in the high FE pigs (Figure 1B). Functional annotation of these DEGs was performed to explore their potential biological roles. Several metabolic processes, including the “fatty acid metabolic process” and “metabolism of lipids”, were significantly enriched among the upregulated genes, suggesting that these genes may contribute to enhanced FE through lipid metabolism-related pathways (Figure 1C). Additionally, the term “enterocyte cholesterol metabolism” was enriched among the downregulated genes, implying that these genes might influence FE by modulating nutrient absorption in the jejunum (Figure 1C). Interestingly, several terms related to viral processes were also enriched in the downregulated genes, suggesting a potential relationship between FE and disease resistance, consistent with previous studies [24,25]. Gene Set Enrichment Analysis (GSEA) on three representative enriched functional terms was further performed to validate our findings, and the GSEA results were consistent with our initial findings, further supporting the functional relevance of these pathways to FE in pigs (Figure 1D) [26,27].

### 3.2. Identification and Characterization of Candidate CREs in the Jejunum of High and Low FE Pigs

**Figure 2:**
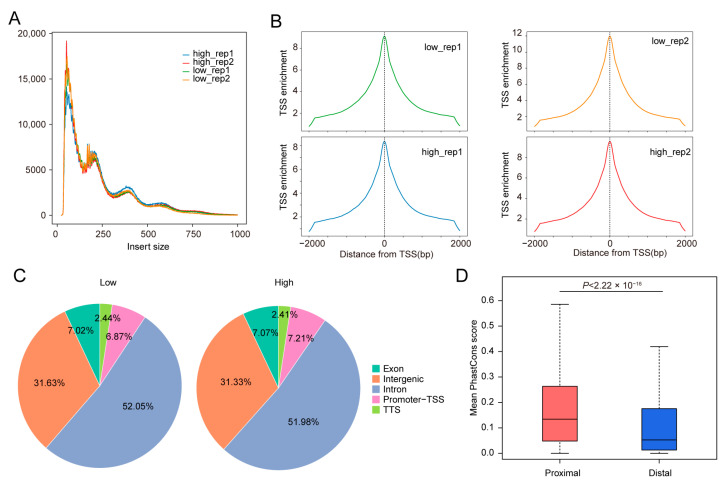
Identification and characterization of cCREs using ATAC-seq data. (**A**,**B**), Insert size distribution and transcription start site (TSS) enrichment analysis of the four sequenced datasets. (**C**), Genomic annotation of candidate cis-regulatory elements (cCREs) identified in high and low FE pigs, respectively. (**D**), Comparison of mean PhastCons conservation scores between proximal and distal cCREs.

Chromatin accessibility plays a critical role in mediating gene expression [28]. To further investigate the regulatory mechanisms associated with FE, we conducted ATAC-seq on the jejunal samples from the four pigs. Following data preprocessing, we obtained 177.94 GB of sequencing data, with an average mapping ratio of 93.55% (Supplementary Table S3). We then assessed the quality of the ATAC-seq data by examining fragment length distribution and transcription start site (TSS) enrichment for each sample. The results demonstrated that the data were of high quality (Figure 2A,B). We also evaluated the reproducibility across the four samples, which revealed high Spearman correlation coefficients (0.91~0.94) between each pair of samples. Additionally, the correlations between replicates of high and low FE pigs were significant (*p* < 2.2 × 10^−16^), with correlation coefficients around 0.94 (Supplementary Figure S2).

To preserve the distinctiveness between high and low FE groups, we identified peaks separately for the two groups, resulting in 189,289 peaks for high FE pigs and 189,251 peaks for low FE pigs. Genomic annotation of the identified peaks revealed that, in both datasets, the majority of peaks were located in intronic regions, followed by intergenic regions, which is consistent with previous studies [29,30]. To further assess the reliability of the identified peaks, we merged the peaks from both groups and classified them into two categories (“Proximal” and “Distal”) based on their distance from the TSS, with “Proximal” referring to peaks less than 2000 bp away from the TSS and “Distal” referring to peaks more than 2000 bp away from the TSS. We then calculated PhastCons scores for each group of peaks. As shown in Figure 2D, the Proximal peaks exhibited significantly higher conservation than the Distal peaks, aligning with previous research [31]. Therefore, in subsequent analyses, we referred to these peaks as candidate cis-regulatory elements (cCREs).

### 3.3. Differential Analysis of Chromatin Accessible cCREs Between Low and High FE Pigs

**Figure 3:**
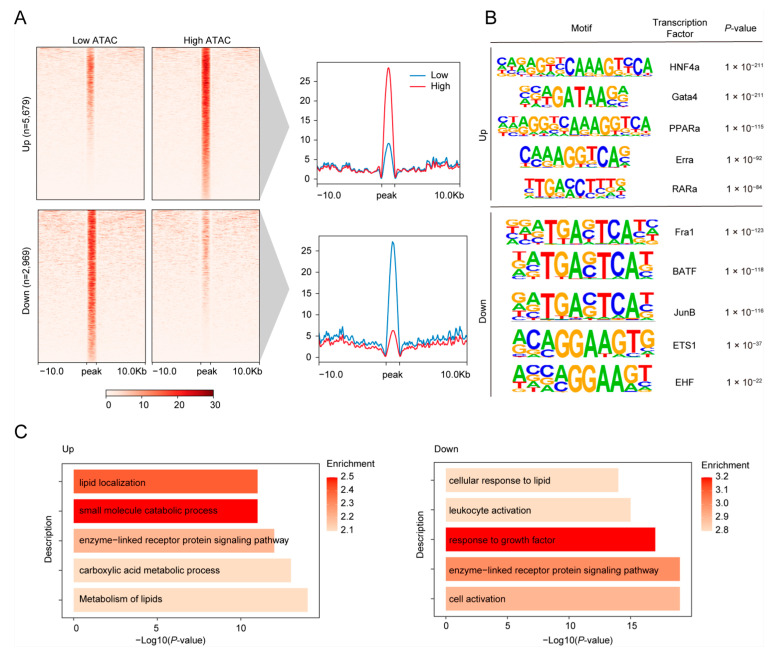
Differential accessibility analysis between high and low FE pigs. (**A**), Heatmaps (**left**) and profile plots (**right**) generated by Deeptools show differentially accessible peaks centered on peak regions. Heatmaps display the read density for each peak, with each row representing a single peak. The comparison of peak accessibility between low and high FE pigs is shown by the mean values of the corresponding peak groups in the right panel. “Up” and “Down” refer to upregulated and downregulated peaks in high FE pigs, respectively. (**B**), Top 5 enriched motifs in the “Up” and “Down” peak groups. (**C**), Functional annotation of target genes associated with peaks in the “Up” and “Down” groups.

Based on our robust ATAC-seq datasets and identified cCREs, we compared the accessibility of cCREs between low and high FE pigs, identifying 5679 upregulated and 2969 downregulated DARs in high FE pigs (Figure 3A).

Building on these findings, we conducted motif enrichment analysis to identify key regulators associated with the DARs. As shown in Figure 3B, the top five enriched motifs for upregulated cCREs included HNF4a, Gata4, PPARa, Erra, and RARa, while the downregulated cCREs were enriched for Fra1, BATF, JunB, ETS1, and EHF. HNF4a is a transcription factor that plays a crucial role in regulating gene expression in various tissues, including the liver, kidney, pancreas, and intestine [32]. In the jejunum, HNF4a can influence lipid metabolism and energy expenditure [33]. GATA4 is essential for regulating gene expression in the jejunum, playing a key role in maintaining intestinal integrity and nutrient absorption. Its activity directly influences FE by enhancing the absorption of nutrients and affecting metabolic pathways related to energy balance [34,35]. EHF plays a crucial role in the jejunum by regulating epithelial cell differentiation and maintaining intestinal barrier integrity, which is vital for nutrient absorption and overall gut health. EHF maintains epithelial homeostasis, regulates cell differentiation in the jejunum, and can optimize nutrient absorption and metabolism, contributing to improved FE in livestock [36,37].

To characterize the biological functions of the DARs, we performed Gene Ontology (GO) annotation using the nearest genes associated with these cCREs. Notably, the terms enriched in upregulated cCREs were linked to lipid metabolic processes, such as “lipid localization” and “metabolism of lipids”, aligning with previous findings from the DEGs (Figure 3C). Conversely, the downregulated cCREs also included lipid-related terms like “cellular response to lipid,” along with “response to growth factor” and “enzyme-linked receptor protein signaling pathway” (Figure 3C). These findings suggest that DARs-regulated cCREs play a crucial role in lipid metabolism, which is directly linked to FE in pigs.

### 3.4. Comparison of Gene Expression and Chromatin Accessibility Revealing High Consistency Between Two Modalities

**Figure 4:**
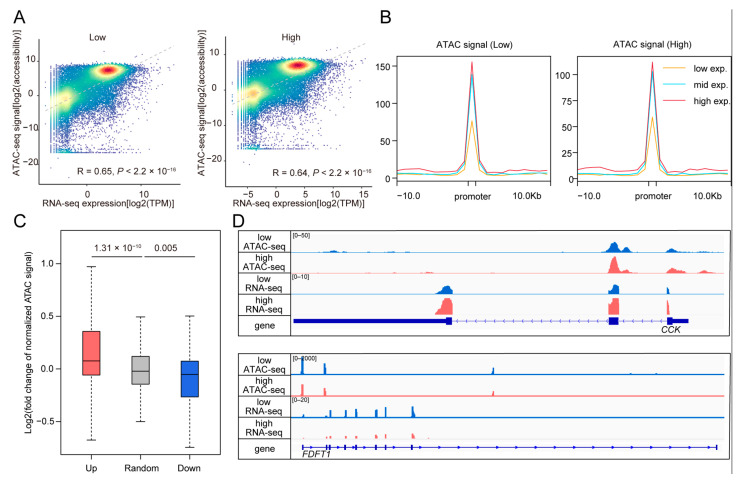
Integrated analysis of RNA-seq and ATAC-seq datasets. (**A**), Density scatter plots illustrating the correlation between gene expression levels and chromatin accessibility at corresponding promoters in low (**left**) and high (**right**) FE pigs, respectively. (**B**), Chromatin accessibility of promoters in three gene expression groups: “low exp.” (<25% quantile), “mid exp.” (25~75% quantile), and “high exp.” (>75% quantile). (**C**), Comparison of promoter chromatin accessibility among genes in the “Up”, “Random”, and “Down” groups. “Up” and “Down” represent genes upregulated and downregulated in high FE pigs, respectively, while “Random” refers to randomly selected genes with no significant expression changes between low and high FE pigs. (**D**), Examples showing consistent changes in gene expression and chromatin accessibility. The upper and lower panels display ATAC-seq and RNA-seq genomic tracks around the *CCK* and *FDFT1* genes that are upregulated and downregulated in high FE pigs, respectively. Genomic tracks from top to bottom display the normalized ATAC-seq reads for low and high FE pigs, RNA-seq reads for low and high FE pigs, and the corresponding gene annotation.

To investigate the relationship between gene expression and chromatin accessibility, we first evaluated the consistency between these two datasets. By calculating the correlations between gene expression levels and chromatin accessibility at the promoters of corresponding genes in both high and low FE pigs, we found significant correlation coefficients in each group (Figure 4A). We then categorized the genes into three groups based on their ranked expression levels: “high” (>75% quantile), “mid” (25–75% quantile), and “low” (<25% quantile). As shown in Figure 4B, chromatin accessibility was markedly decreased in the “low” group compared to the other groups, while the “mid” group exhibited slightly lower accessibility than the “high” group. These results indicate a strong correlation between gene expression and chromatin accessibility, supporting further comparisons between the two datasets.

Next, we compared chromatin accessibility among upregulated, downregulated, and randomly selected genes, revealing significant consistency (Figure 4C). Two examples, *CCK* and *FDFT1*, are illustrated in Figure 4D. *CCK* exhibited significant upregulation in both gene expression and chromatin accessibility, while *FDFT1* showed downregulation in high FE pigs. *CCK* is a gut hormone that regulates digestion by stimulating the release of digestive enzymes and bile, and promoting satiety, enhancing nutrient digestion and absorption [38]. *FDFT1* encodes squalene synthase, an evolutionarily conserved enzyme and a key regulator of cholesterol synthesis [39].

### 3.5. Integrating DEGs and DARs to Construct a FE-Related Regulatory Gene Network

**Figure 5:**
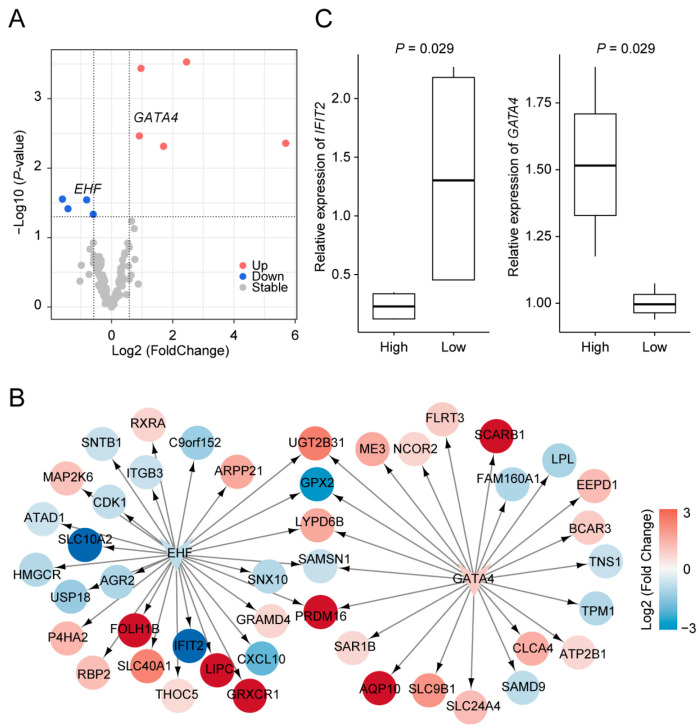
Regulatory gene network related to feed efficiency (FE). (**A**), Comparison of transcription factor expression between low and high FE pigs. (**B**), The regulatory gene network is constructed using selected transcription factors and their target genes. All transcription factors and genes included in the network show significant differential expression between low and high FE pigs. The color bar represents the log2 fold changes in gene expression for high versus low FE pigs. (**C**), Box plots illustrate the relative expression of *IFIT2* and *GATA4* genes, quantified by qPCR.

FE is a complex trait involving multiple genes and regulated by intricate networks. To construct a regulatory gene network related to FE, we integrated our findings from gene expression and chromatin accessibility analyses. We first identified common genes that were DEGs and acted as transcription factors enriched in DARs. This analysis yielded five upregulated and four downregulated transcription factors in high FE pigs (Figure 5A). Notably, two transcription factors, EHF and GATA4, are associated with FE.

Based on EHF and GATA4, we constructed a regulatory network that includes target genes significantly altered in gene expression between high and low FE pigs. Among these, five genes—*UGT2B31*, *GPX2*, *LYPD6B*, *SAMSN1*, and *PRDM16*—are regulated by both EHF and GATA4. *PRDM16* is involved in maintaining the homeostasis of the intestinal epithelium by controlling region-specific metabolism and epithelial renewal. *PRDM16* influences fatty acid oxidation (FAO) in crypt-resident progenitor cells, which is crucial for developing and maintaining intestinal enteroids [40]. In addition to these five genes, EHF regulates 23 other genes, and GATA4 regulates 17. Among these, we identified several additional genes linked to FE, including *SCARB1*, which is involved in intestinal absorption of plasma triglyceride (TG), and *GRXCR1*, related to residual feed intake (RFI) [41,42]. Including these genes further emphasizes the role of EHF and GATA4 in modulating metabolic pathways related to FE.

## 4. Discussion

FE is a complex trait influenced by various factors, including genetic and environmental factors [43,44]. The jejunum, as a critical organ for nutrient absorption, plays a pivotal role in determining FE [4,5]. By integrating RNA-seq and ATAC-seq data, our study aimed to identify key genetic and epigenetic mechanisms underlying jejunal function and its impact on FE.

Our analysis revealed that DEGs and DARs were enriched in pathways related to lipid metabolism and immune response. These findings suggest that the jejunum plays a crucial role in regulating lipid metabolism and immune function, both of which are closely linked to FE. Furthermore, we identified two key transcription factors, EHF and GATA4, as potential regulators of FE. These transcription factors may coordinate the expression of genes involved in nutrient absorption, metabolism, and immune function, ultimately impacting FE.

While this study provides valuable insights into the molecular mechanisms underlying FE, it is important to acknowledge its limitations. Our analysis was focused on the jejunum, and a more comprehensive understanding of FE would require investigating other tissues, such as the tissues in the gut–brain axis, which has been reported to involve in FE [45,46]. Additionally, future studies could explore the interactions between genetic and environmental factors that influence FE.

Our study provides a comprehensive analysis of the molecular mechanisms underlying FE in the jejunum. By identifying key genes and transcription factors, we have laid the foundation for further research to develop strategies to improve FE in pigs. Future research should focus on validating these findings in larger populations and exploring the potential of genetic interventions to enhance FE.

## 5. Conclusions

Our study sheds light on the molecular basis of feed efficiency (FE) in pigs, particularly focusing on the role of the jejunum in nutrient absorption. By conducting an integrative multi-omics analysis using RNA-seq and ATAC-seq, we identified key genes, chromatin regions, and regulatory factors associated with lipid metabolism and immune response that likely influence FE. Transcription factors like GATA4 and EHF, along with genes such as *SCARB1* and *GRXCR1*, emerged as potential regulators of efficient feed use. These findings offer valuable molecular targets for enhancing FE through genetic selection and tailored nutritional strategies. Ultimately, this research contributes to more sustainable and economically viable pig farming practices, aligning with goals for environmentally responsible agriculture.

## Data Availability

The original data presented in the study are openly available in the NCBI Gene Expression Omnibus database with accession number GSE282017.

## Supplementary Materials

The following supporting information can be downloaded at www.mdpi.com/article/10.3390/ani15020137/s1. Table S1. Overview of sequenced individuals. Table S2: Summary of RNA-seq data for each sample; Figure S1: Assessment of RNA-seq data reproducibility; Table S3: Summary of ATAC-seq data for each sample; Figure S2: Assessment of ATAC-seq data reproducibility.
