# Supplementary material for: Uncovering Molecular Mechanisms of Feed Efficiency in Pigs Through Multi-Omics Analysis of the Jejunum
Source: Animals (Basel). 2025 Jan 8;15(2):137. doi: 10.3390/ani15020137 (PMC11758640; doi:10.3390/ani15020137)
Supplement: Supplementary file 1 [file animals-15-00137-s001.zip › Supplementay Figures.pdf]

Supplementary Figures

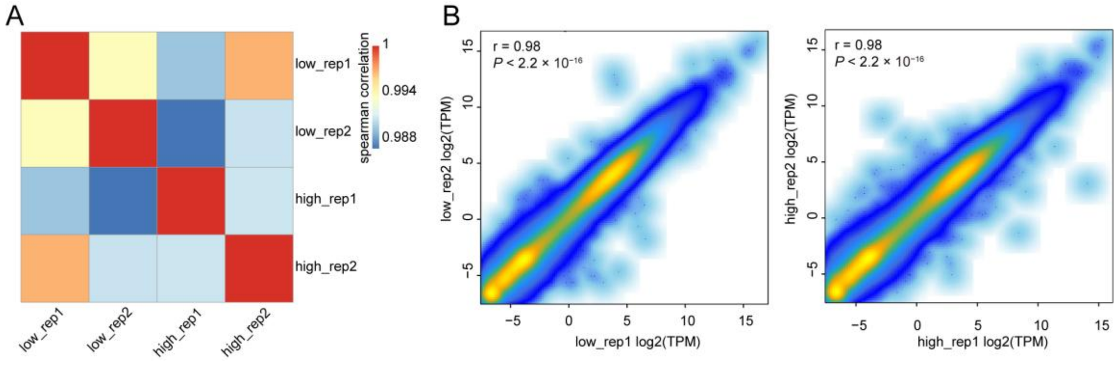

Supplementary Figure S1. Assessment of RNA-seq data reproducibility. A, Spearman correlation coefficients between each pair of the four sequenced datasets. B, Density scatter plots illustrating the correlation between replicates within low FE (left) and high FE (right) pigs, respectively.

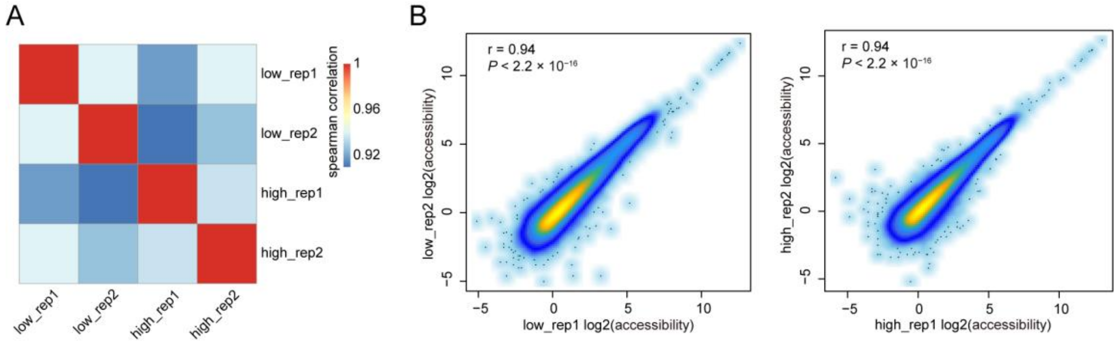

Supplementary Figure S2. Assessment of ATAC-seq data reproducibility. A, Spearman correlation coefficients between each pair of the four sequenced datasets. B, Density scatter plots illustrating the correlation between replicates within low FE (left) and high FE (right) pigs, respectively.
