# Supplementary material for: Uncovering Molecular Mechanisms of Feed Efficiency in Pigs Through Multi-Omics Analysis of the Jejunum
Source: Animals (Basel). 2025 Jan 8;15(2):137. doi: 10.3390/ani15020137 (PMC11758640; doi:10.3390/ani15020137)
Supplement: Supplementary file 1 [file animals-15-00137-s001.zip › Supplementay Tables.pdf]

Supplementary Table S1. Overview of sequenced individuals.

Sample	FCR	Breed	Gender	Body weight (kg)
Low 1	3.42	Large White	M	101.00
Low 2	2.88	Large White	M	101.75
High 1	2.11	Large White	M	102.50
High 2	2.21	Large White	M	100.00

Supplementary Table S2. Summary of RNA-seq data for each sample.

Sample	Reads count	Base pairs	Q20 (%)	Q30 (%)	GC content (%)	Ratio of uniquely mapped reads (%)
Low 1	95,563,758	14,253,524,476	96.39	91.12	47.36	86.50%
Low 2	82,087,398	12,244,532,556	96.60	91.50	48.26	84.58%
High 1	92,855,378	13,849,363,963	97.06	92.37	49.90	82.85%
High 2	79,898,456	11,913,873,040	97.25	92.69	47.73	87.43%

Supplementary Table S3. Summary of ATAC-seq data for each sample.

Sample	Reads count	Base pairs	Q20 (%)	Q30 (%)	GC content (%)	Mapping ratio (%)
Low 1	405,909,050	47,259,631,302	96.6	91.75	48.55	93.20
Low 2	420,770,626	48,220,082,682	96.44	91.48	49.89	94.00
High 1	316,854,886	38,062,397,375	95.06	87.93	47.84	93.80
High 2	389,952,074	44,403,977,119	96.28	91.26	48.67	93.20
